# Comparison of Wavelight Allegretto Eye-Q and Schwind Amaris 750S excimer laser in treatment of high astigmatism

**DOI:** 10.1007/s00417-014-2776-2

**Published:** 2014-08-23

**Authors:** Maja Bohac, Alma Biscevic, Mateja Koncarevic, Marija Anticic, Nikica Gabric, Sudi Patel

**Affiliations:** 1 Specialty Eye Hospital „Svjetlost“, Heinzelova 39, 10000 Zagreb, Croatia; 2 NHS National Services Scotland, Edinburgh, UK

**Keywords:** Astigmatism, LASIK, Excimer laser, Ablation profile, High-order aberrations

## Abstract

**Purpose:**

To compare functional outcomes of Wavelight Allegretto Eye-Q 400Hz and Schwind Amaris 750S excimer laser for astigmatism between 2 and 7 diopters(D).

**Methods:**

Prospective comparative non-randomized case series of 480 eyes assigned in two laser groups and further divided into myopic and mixed astigmatism subgroups. All treatments were centered on corneal vertex. One-year results were compared between the groups. Statistical analysis was performed using z-test.

**Results:**

Both Allegretto and Amaris postoperative uncorrected distance visual acuity (UDVA) improved in comparison to preoperative corrected distance visual acuity (CDVA). The difference was significant in the Allegretto group for myopic astigmatism (*p* = 0.017). There was no difference in postoperative UDVA between lasers. Average sphere decreased in all groups for both lasers (p < 0.001) without difference in effectiveness of spherical correction between lasers for both groups. In Allegretto, average cylinder decreased from −3.30D to −0.55D in myopic astigmatism (*p* < 0.001) and from −3.84D to −0.85D in mixed astigmatism (*p* < 0.001). In Amaris average cylinder decreased from −3.21D to −0.43D in myopic astigmatism (*p* < 0.001) and from −3.66D to −0.58D in mixed astigmatism (*p* < 0.001). Amaris group had less residual astigmatism (myopic astigmatism *p* = 0.023, mixed astigmatism *p* < 0.001). Mean spherical aberration shifted from positive to negative in mixed astigmatism for both lasers.

**Conclusion:**

Both lasers are effective in terms of UDVA, CDVA, spherical correction, and preservation of high-order aberrations. However, Amaris was more effective in cylinder correction.

## Introduction

LASER in-situ keratomileusis (LASIK) is a highly successful keratorefractive procedure for the treatment of myopia and low degrees of hyperopia. However, treatment of astigmatism, especially hyperopic astigmatism, is still a therapeutic challenge, and often results in significant refractive misscorrections [1, 2] . Recent advances in excimer laser technology, such as the use of aspheric ablation profiles, incorporation of higher-order aberration treatment, and eye trackers, have presumably led to better refractive outcomes and reduced induction of higher-order aberrations postoperatively [3, 4].

The Wavelight Allegretto Eye-Q is a flying-spot excimer laser, with a pulse repetition rate of 400Hz, with two galvanometric scanners for positioning laser pulses. The beam is a small-spot, <0.95 mm in diameter, with a Gaussian energy distribution. The system has an infrared high-speed camera operating at 400Hz to track the patient’s eye movements that either compensates for changes in eye position or interrupts the treatment if the eye moves outside a preset predetermined range. The Wavelight Allegretto Eye-Q laser delivery program is designed to maintain a more natural corneal shape by adjusting for the asphericity of the cornea based on the anterior curvature readings, and minimizing the amount of spherical aberration induced during surgery [5, 6]. The system compensates for the slope in the cornea by delivering a relatively larger number of pulses to the periphery. The Schwind Amaris 750S is a flying-spot excimer laser with a pulse repetition rate of 750Hz, spot diameter of 0.54 mm, and Gaussian energy distribution. The Schwind Amaris 750S laser delivery program also features an aspheric ablation algorithm for refractive treatments. Depending on the planned refractive correction, approximately 80 % of the corneal ablation is performed with a high fluence level (>400 mJ/cm^2^) and this leads to a considerable reduction in time spent treating the cornea. Fine correction is performed for the remaining 20 % of the treatment using a low fluence level (<200 mJ/cm^2^), aimed to reduce the amount ablated per pulse and smooth out the ablated stromal bed. The laser features a five-dimensional 1050Hz infrared eye tracker with simultaneous limbus, pupil, iris recognition, and cyclotorsion tracking integrated in the laser delivery process. One of the optional ablation algorithms is the “Aberration- Free^TM^” package that is designed to maintain the preoperative levels of ocular higher-order aberrations [7–10].

The aim of the present study was to investigate and compare refractive, visual, and optical results after treatment of high astigmatism by these two lasers.

## Materials and methods

This was a prospective non randomized consecutive comparative case series approved by the Ethics Committee at “Svjetlost” Specialty Eye Hospital. The tenets of the Helsinki agreement were followed throughout.

Between January 2010 and December 2011, 470 eyes (274) patients with astigmatism more than 2 diopters (D) were operated in “Svjetlost” Specialty Eye Hospital in Zagreb, Croatia. 418 eyes (237 patients) completed one year follow up. Only the eyes that completed 1-year follow-up were included in this study. The eyes were divided in two groups according to the laser platform on which were treated — Wavelight Allegretto Eye-Q 400Hz and Schwind Amaris 750S. Within each group, the treated eyes were further subdivided according to the type of astigmatism, myopic astigmatism, or mixed astigmatism. A total of 188 eyes (110 patients) were included in the Allegretto group. There were 127 eyes (71 patients) with myopic astigmatism and 61 eyes (39 patients) with mixed astigmatism. A total of 230 eyes (127 patients) were included in the Amaris group. There were 119 eyes (64 patients) with myopic astigmatism and 111 eyes (63 patients) with mixed astigmatism.

### Patient allocation

Each patient was assigned to a particular laser group by administrative staff according to the patient’s scheduling needs and availability of technical teams required for each laser. Although this is not a true randomization process, the surgeon performing the treatment had no influence on patient assignment to the groups.

### Preoperative examinations

All patients underwent a complete preoperative ophthalmologic examination prior to deciding if the patient met the criteria for surgery. Patients with stable refraction, astigmatism  ≥2.0D, regardless of the amount of myopic or hyperopic spherical correction, were included. Ocular criteria were those normally adopted in refractive surgery. Patients with history of ocular surgery, abnormal corneal topography, preoperative corneal thickness  <490 μm or calculated residual stromal bed thickness <280 μm were excluded from the study.

Examination included uncorrected distance visual acuity (UDVA), corrected distance visual acuity (CDVA), manifest and cycloplegic refraction, corneal topography (Pentacam HR^TM^, Oculus Optikgeräte GmbH, Wetzlar, Germany), aberrometry (L 80 wave + ^TM^, Luneau SAS, Prunay-le-Gillon, France), tonometry (Auto Non-Contact Tonometer, Reichert Inc., Buffalo, NY, USA), slit-lamp and dilated funduscopic examination. Visual acuity was measured using a standard Snellen acuity chart at 6 m, and presented in decimal format.

The patients were asked to discontinue contact lens wear for up to 4 weeks, depending on the type of contact lenses, prior to the examination.

### Surgical procedure and postoperative care

Prior to the surgery, two drops of topical anesthetic (Novesin, OmniVision GmbH, Puchheim, Germany) were instilled at 2-minute intervals, and the eye was cleaned with 2.5 % povidone iodine. A corneal flap was cut using Moria M2 mechanical microkeratome with 90 μm head (Moria, Antony, France). Either Wavelight Allegretto Eye-Q 400Hz (Alcon, Forth Worth, TX, USA) or Schwind Amaris 750S (Schwind eye-tech-solutions, Kleinostheim, Germany) were used for the excimer laser treatment. In all patients treated with Allegretto Eye-Q laser, the optical zone was fixed at 6.5 mm as recommended by the manufacturer, and the wavefront optimized program was used. Since the laser ablation algorithm is based on preservation of corneal asphericity by delivering additional laser pulses on the periphery to maintain a natural corneal shape, total ablation zones were wide; 8.9 mm for mixed astigmatism and 9.0 mm for myopic astigmatism cases. For the Amaris 750S, the mean optical zone of the treatment was 6.63 ± 0.20 mm (range 6.5 to 7.0 mm). The rationale for changing optical zone was based on the manufacturer’s recommendation to select, at least, a 6.7 mm optical zone for treatment of astigmatism. However, we did not want to exceed 9.0 mm zone of total ablation. Since the transition zone (automatically calculated by the system for the selected optical zone and applied correction) increases with the complexity of the applied correction, the size of an optical zone was chosen to fit within the limits of a 9.0 mm of total ablation zone. The total ablation zone was 8.67 ± 0.31 mm (range 7.9 to 9.0 mm). The Aberration Free^TM^ program was applied in all cases.

All ablations were centered on corneal vertex for both laser platforms. The corneal vertex is the intersection of the pupillary axis with the anterior surface of the cornea, when the pupillary axis coincides with the optical axis of the measuring device [11]. The position of the corneal vertex was determined by the pupillary offset, that is the distance between the pupil center and the normal corneal vertex [12], calculated by using the videokeratoscope (CSO, Costruzione Strumenti Oftalmici, Florence, Italy) for Amaris, and Scheimpflug camera (Pentacam HR^TM^, Oculus Optikgeräte GmbH, Wetzlar, Germany) for Allegretto. The Cartesian coordinates of the corneal vertex were manually entered into the software program [13]. For all patients, the programmed treatment consisted of cycloplegic spherical correction with manifest astigmatic power and axis. For the Allegretto Eye-Q, the “Wellington nomogram” provided by the company was used for spherical correction. The nomogram also directs the surgeon to correct 25 % less of the full astigmatism. Our previous experience (unpublished) showed that the 25 % modification led to significant undercorrection. Thus, we decided to use an empirically derived undercorrection of 15 % in all cases where the Allegretto Eye-Q was used. For the Amaris 750S the sphere, cylinder, and axis were entered into laser without nomogram adjustment. Before excimer laser ablation, proper alignment of the eye with Allegretto Eye-Q was achieved with a manual cross technique to compensate for cyclotorsion. When Amaris 750S was used for treatment, the built-in eye tracker automatically compensated for static and dynamic cyclotorsion of the eye. In all cases, the flap was lifted and excimer laser ablation was delivered to the stroma. Patients were instructed to concentrate on the fixation light throughout the ablation. When the ablation was completed, the flap was repositioned after the interface was irrigated with balanced salt solution, removing any debris.

Postoperative therapy included combination of topical antibiotic and steroid drops (Tobradex, Alcon, Forth Worth, TX, USA) 4 times daily for 2 weeks, and artificial tears (Blink, Abbott Medical Optics, Santa Ana, CA, USA) 6–8 times daily for at least 1 month.

### Postoperative evaluation

All patients were examined 1 day, 1 week, 1 month, 3 months, and 1 year after the surgery. Results 1 year after the surgery were analyzed in this study. Evaluation included measurement of UDVA, CDVA, manifest refraction, aberrometry, slit-lamp examination, tonometry, and corneal topography.

### Data and statistical analysis

Data were analyzed to determine significance of change in spherical correction, astigmatism, UDVA, CDVA, high-order aberrations, (2-sample z-test assuming unequal variances for data with normal distribution, and Mann–Whitney U test for non-parametric analysis) within each group and between groups, to determine whether there was significant difference between two lasers. Changes and differences were considered statistically significant when *p* < 0.05. Spherical aberration was further analyzed with cluster analysis to see the trend in change.

Data were further analyzed with Pearson correlation to determine the significance of any correlation between pre- and postoperative sphere, cylinder, and visual acuity. Correlations were considered significant when *p* < 0.05.

## Results

Both laser groups were well-balanced, and there was no statistically significant difference preoperatively between the groups in terms of UDVA, CDVA, sphere, cylinder, and amount of high-order aberrations. 57 males (52 %) and 52 females (48 %) were treated on Allegretto, while 71 males (56 %) and 56 females (44 %) were treated on Amaris. Avarage age (years ± SD) of the patients was 32.69 ± 7.2 (range 20–58) for the Allegretto, and 33.25 ± 6.9 (range 20–58) for the Amaris group.

### Visual acuity

In the Allegretto group, values of postoperative UDVA showed improvement in comparison to preoperative CDVA. The difference was 0.5 Snellen lines in myopic astigmatism, and was significant (*p* = 0.017), while in mixed astigmatism the difference was 0.3 Snellen lines and was not statistically significant (*p* = 0.406). In the Amaris group, improvement in postoperative UDVA in comparison to preoperative CDVA was observed, but improvement of 0.5 Snellen lines was not significant for the myopic astigmatism group (*p* = 0.06), neither were 0.3 Snellen lines significant for the mixed astigmatism group (*p* = 0.115) (Tables 1 and 2). There was no difference in postoperative UDVA between lasers for myopic astigmatism (*p* = 0.967) and for mixed astigmatism Amaris was better for 0.3 Snellen lines but without statistical significance (*p* = 0.151). None of the eyes lost any lines of CDVA.

**Table 1:** Comparison of visual and refractive results at 1 year with baseline values in myopic astigmatism showing the main results of the investigation

Visual and refractive results — myopic astigmatism						
Mean ± standard deviation						
Variable	Wavelight Allegretto Eye-Q			Schwind Amaris 750S		
	preop	postop	*p* value*	preop	postop	*p* value*
UDVA	0.15 ± 0.15 (0.01 to 0.70)	0.86 ± 0.16 (0.35 to 1.00)	<0.001	0.13 ± 0.11 (0.01 to 0.45)	0.86 ± 0.19 (0.15 to 1.00)	<0.001
CDVA	0.81 ± 0.17 (0.30 to 1.00)	0.89 ± 0.16 (0.40 to 1.00)	<0.001	0.81 ± 0.18 (0.10 to 1.00)	0.89 ± 0.20 (0.20 to 1.00)	0.001
sphere (D)	−2.80 ± 2.01 (−8.50 to 0.00)	−0.16 ± 0.46 (−1.50 to 1.00)	<0.001	−2.44 ± 2.17 (−7.50 to 0.00)	−0.16 ± 0.55 (−2.00 to 1.25)	<0.001
cylinder (D)	−3.30 ± 1.00 (−7.50 to −2.00)	−0.55 ± 0.46 (−2.25 to 0.00)	<0.001	−3.21 ± 0.87 (−6.50 to −2.00)	−0.43 ± 0.36 (−1.50 to 0.00)	<0.001

UDVA = uncorrected distance visual acuity (decimal)

CDVA = corrected distance visual acuity (decimal)

D = diopter

*2-sample z-test assuming unequal variances

**Table 2:** Comparison of visual and refractive results at 1 year with baseline values in mixed astigmatism showing the main results of the investigation

Visual and refractive results — mixed astigmatism						
Mean ± standard deviation						
Variable	Wavelight Allegretto Eye-Q			Schwind Amaris 750S		
	preop	postop	*p* value*	preop	postop	*p* value*
UDVA	0.25 ± 0.14 (0.03 to 0.60)	0.77 ± 0.20 (0.20 to 1.00)	<0.001	0.24 ± 0.12 (0.02 to 0.60)	0.80 ± 0.21 (0.05 to 1.00)	<0.001
CDVA	0.74 ± 0.22 (0.15 to 1.00)	0.82 ± 0.21 (0.20 to 1.00)	0.04	0.77 ± 0.20 (0.04 to 0.95)	0.85 ± 0.21 (0.10 to 1.00)	0.004
sphere (D)	2.72 ± 1.79 (0.25 to 7.00)	0.19 ± 0.52 (−1.50 to +1.50)	<0.001	3.11 ± 1.57 (0.50 to 7.50)	0.28 ± 0.45 (−0.75 to 1.00)	<0.001
cylinder (D)	−3.84 ± 1.21 (−6.50 to −2.00)	−0.85 ± 0.41 (−2.00 to 0.00)	<0.001	−3.66 ± 1.16 (−7.00 to −2.00)	−0.58 ± 0.38 (−1.50 to 0.00)	<0.001

UDVA = uncorrected distance visual acuity (decimal)

CDVA = corrected distance visual acuity (decimal)

D = diopter

*2-sample z-test assuming unequal variances

### Refraction

In the Allegretto group, significant decrease of sphere and cylinder was observed for both the myopic and mixed astigmatism groups. Average sphere decreased from −2.80D to −0.16D in the myopic astigmatism group (*p* < 0.001) and from +2.72D to +0.19D in the mixed astigmatism group (*p* < 0.001). Average cylinder decreased from −3.30D to −0.55D in the myopic astigmatism group (*p* < 0.001), and from −3.84D to −0.85D in the mixed astigmatism group (*p* < 0.001) (Tables 1 and 2).

In the Amaris group, significant decrease of sphere and cylinder was observed for both the myopic and mixed astigmatism groups. Average sphere decreased from −2.44D to −0.16D in the myopic astigmatism group (*p* < 0.001) and from +3.11D to +0.28D in the mixed astigmatism group (*p* < 0.001) Average cylinder decreased from −3.21D to −0.43D in the myopic astigmatism group (*p* < 0.001), and from −3.66D to −0.58D in the mixed astigmatism group (*p* < 0.001) (Tables 1 and 2).

**Fig. 1:**
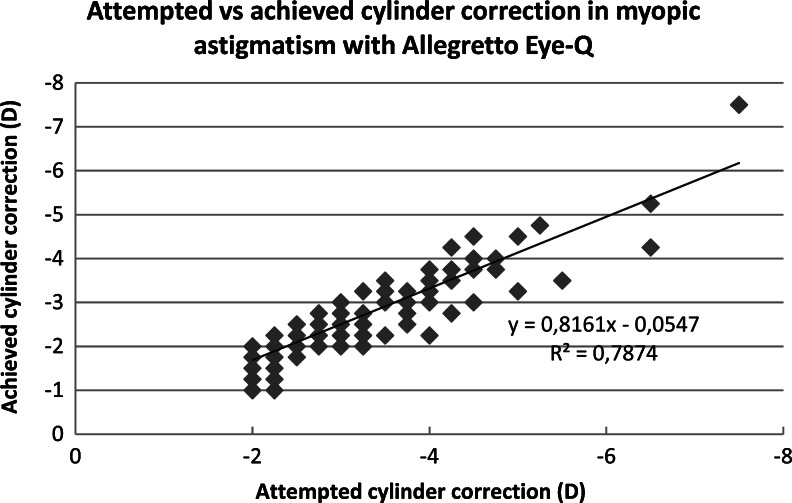
Attempted vs achieved cylinder correction (D) in myopic astigmatism with Allegretto Eye-Q. The least-squares regression line, best-fit linear equation and R2 are included for comparison with Figs. 2, 3, & 4

**Fig. 2:**
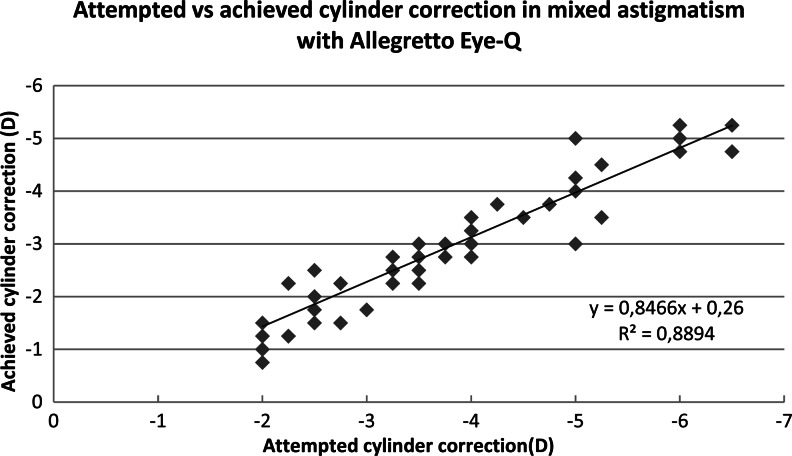
Attempted vs achieved cylinder correction (D) in mixed astigmatism with Allegretto Eye-Q. The least-squares regression line, best-fit linear equation and R2 are included for comparison with Figs. 1, 3, & 4

**Fig. 3:**
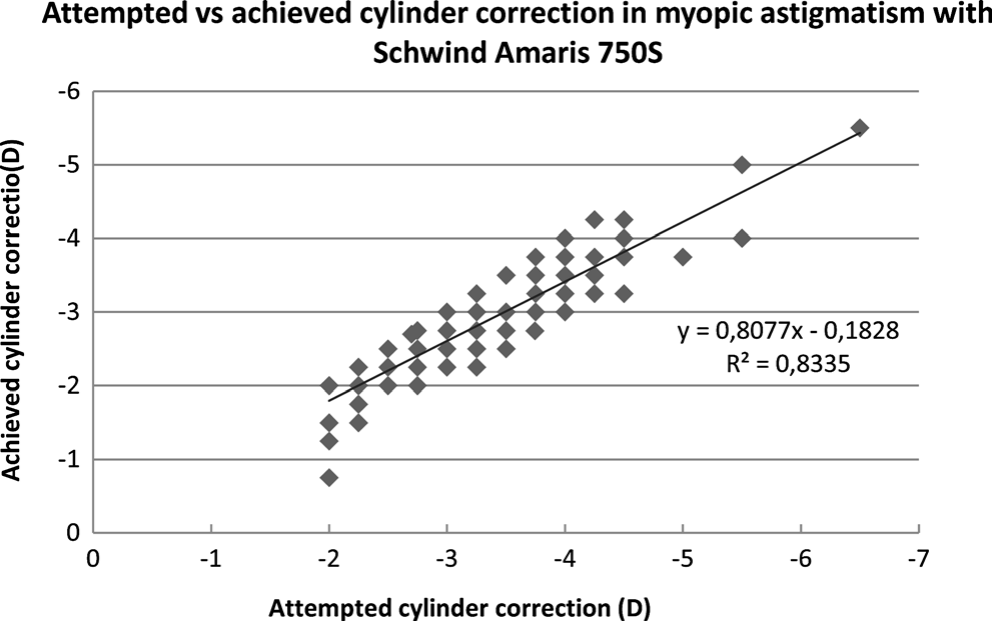
Attempted vs achieved cylinder correction (D) in myopic astigmatism with Amaris 750S. The least-squares regression line, best-fit linear equation and R2 are included for comparison with Figs. 1, 2 & 4

**Fig. 4:**
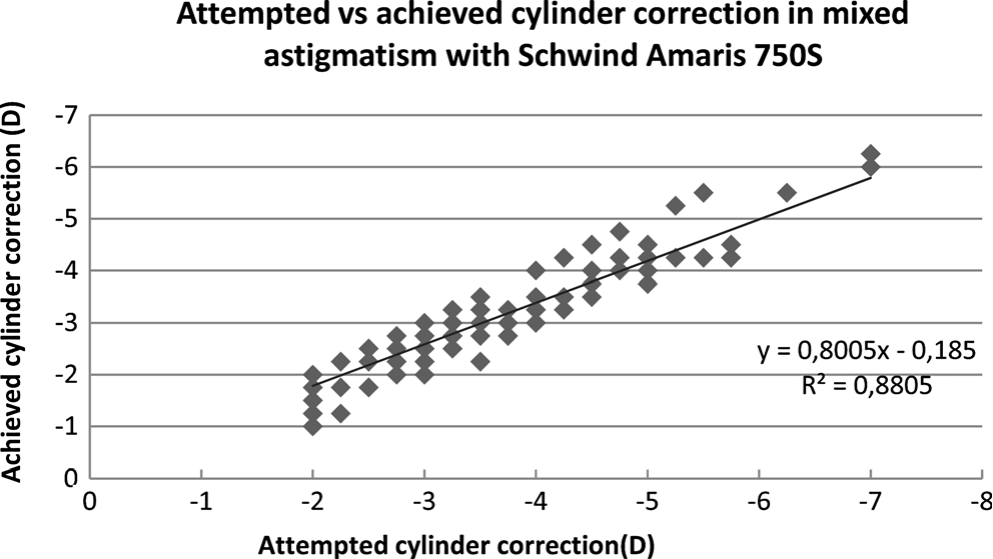
Attempted vs achieved cylinder correction (D) in mixed astigmatism with Amaris 750S. The least-squares regression line, best-fit linear equation and R2 are included for comparison with Figs. 1, 2 & 3

There was no difference in effectiveness of spherical correction between laser platforms for both myopic and mixed astigmatism (*p* = 0.969, *p* = 0.236). The Amaris group had less residual astigmatism than the Allegretto group, and the difference was significant (myopic astigmatism *p* = 0.027, mixed astigmatism *p  *< 0.001). The attempted and achieved astigmatic corrections are shown in Figs. 1, 2, 3, and 4. In all cases, there was a highly significant association between the attempted and achieved astigmatic corrections. For the Allegretto cases, there was a tendency toward residual cylinder in both the myopic and mixed astigmatism groups when comparing the attempted cylindrical correction with the postoperative cylinder (*r * = 0.3978, *p* < 0.01, *n* =127 for myopic, and *r* = 0.4567, *p* < 0.01, *n* = 61 for mixed astigmatism). Similar trends were also found in the Amaris cases (*r *= 0.4701, *p* < 0.01, *n* =119 for myopic, and *r* = 0.3257, *p* < 0.01, *n  *= 111 for mixed astigmatism).

### Aberrometry

In the Allegretto group, there was no significant change between preoperative and postoperative high-order aberrations in the myopic astigmatism group (coma 0.592, trefoil *p* = 0.999, SA *p * = 0.056). Although there was no difference in SA between preoperative and postoperative values, SA showed a tendency to shift towards more positive values in 54.4 % of eyes, and in 42.4 % of eyes showed a tendency to shift towards negative values, while 3.2 % of eyes remained unchanged. In the mixed astigmatism group, there was no significant change in coma and trefoil (*p* = 0.347, *p  *= 0.116) ,while spherical aberration shifted from positive to negative values (*p* = 0.03) (Tables 3 and 4). SA showed a tendency to shift towards negative values in 63.3 % of eyes, and in 26.7 % of eyes showed a tendency to shift towards more positive values, while 10.0 % of eyes remained unchanged.

**Table 3:** Comparison of high-order aberrations (μm) on 5 mm pupil at 1 year with baseline values in myopic astigmatism, showing the main results of the investigation

High-order aberrations — myopic astigmatism						
Variable	Mean ± standard deviation					
	Wavelight Allegretto Eye-Q			Schwind Amaris 750S		
	preop	postop	*p* value*	preop	postop	*p* value*
coma (μm)	0.12 ± 0.09 (0.01 to 0.53)	0.11 ± 0.10 (0.01 to 0.90)	0.592	0.11 ± 0.08 (0.00 to 0.40)	0.13 ± 0.11 (0.01 to 0.80)	0.166
trefoil (μm)	0.11 ± 0.06 (0.01 to 0.32)	0.11 ± 0.13 (0.01 to 0.90)	0.999	0.10 ± 0.06 (0.01 to 0.29)	0.09 ± 0.08 (0.01 to 0.49)	0.211
SA (μm)	−0.02 ± 0.07 (−0.28 to 0.20)	0.00 ± 0.05 (−0.40 to 0.22)	0.056	−0.01 ± 0.05 (−0.23 to 0.10)	−0.01 ± 0.07 (−0.26 to 0.14)	0.504

SA = Spherical aberration (μm – micrometer)

*2-sample z-test assuming unequal variances

**Table 4:** Comparison of high-order aberrations (μm) on 5 mm pupil at 1 year with baseline values in mixed astigmatism, showing the main results of the investigation

High-order aberrations — mixed astigmatism						
Variable	Wavelight Allegretto Eye-Q			Schwind Amaris 750S		
	preop	postop	*p* value*	preop	postop	*p* value*
coma (μm)	0.12 ± 0.08 (0.02 to 0.45)	0.10 ± 0.06 (0.04 to 0.28)	0.347	0.12 ± 0.09 (0.01 to 0.46)	0.11 ± 0.03 (0.01 to 0.53)	0.420
trefoil (μm)	0.13 ± 0.13 (0.01 to 0.90)	0.10 ± 0.05 (0.02 to 0.26)	0.116	0.10 ± 0.07 (0.01 to 0.44)	0.09 ± 0.08 (0.01 to 0.76)	0.404
SA (μm)	0.02 ± 0.06 (−0.11 to 0.14)	0.00 ± 0.04 (−0.09 to 0.12)	0.03	0.02 ± 0.05 (−0.07 to 0.20)	0.00 ± 0.04 (−0.18 to 0.10)	<0.001

SA = Spherical aberration (μm – micrometer)

*2-sample z-test assuming unequal variances

In the Amaris group, there was no significant change between preoperative and postoperative high-order aberrations in the myopic astigmatism group (coma *p* = 0.166, trefoil *p* = 0.211, SA = 0.504). Although there was no difference in SA between preoperative and postoperative values, SA showed a tendency to shift towards more positive values in 41.5 % of eyes, and in 50.0 % showed a tendency to shift towards negative values, while 8.5 % of eyes remained unchanged. In the mixed astigmatism group, there was no significant change in coma and trefoil (*p* = 0.420, *p *= 0.404) ,while spherical aberration shifted from positive to negative values (*p * < 0.001) (Tables 3 and 4). SA showed a tendency to shift towards negative values in 66.1 % of eyes, and in 29.4 % of eyes showed a tendency to shift towards more positive values, while 4.5 % of eyes remained unchanged.

There was no significant difference in the magnitude of high-order aberrations between the lasers for both the myopic astigmatism (coma *p* = 0.137, trefoil *p  *= 0.143, SA *p * = 0.2) and mixed astigmatism groups (coma *p* = 0.222, trefoil *p * = 0.314, SA *p* = 1.00).

## Discussion

The main limitations of our study were its comparative rather than randomized study design, and distinct differences between software and hardware of two excimer lasers in terms of optical zone selection and eye-tracking devices.

As far as patient selection, there was no selection bias with respect to assignment of patients to each laser group, as both lasers were engaged in refractive surgery according to organizational schedules and availability of technical teams needed for each laser. This resulted in patient assignment to laser group by administrative staff according to patient scheduling needs. We understand that this is not a true randomization process. Nevertheless, the surgeon performing the treatments had no influence on patient assignment to the groups, and therefore the bias was minimized.

### Visual acuity

There was an improvement in UDVA and no loss in CDVA for both lasers. In one subgroup, we found post-op UDVA was better than pre-op CDVA. Stonecipher and Kezarian [5] found no loss of CDVA at 6 months using the Allegretto 400Hz platform. Alió et al. [14] using the Amaris 500 platform with an aspheric profile reported 16 % of cases lost up to 1 line on CDVA.

### Refraction

Correction of the sphere was very acceptable for both lasers; however, there was a tendency towards residual cylinder. When analyzing eyes with myopic astigmatism, 48 % of cases were within ±0.50D of intended refraction in the Allegretto group, in comparison to 54 % in the Amaris group (*p * = 0.368). Our results differ from those of Stonecipher et al. [6], who reported 94 % out of 186 cases treated with Wavelight Allegretto 400Hz within ±0.50D of intended refraction. Alió et al. [14] used the Amaris 500 platform with an aspheric profile, and reported a predictability of 87 %. However, those data were based on 37 eyes, whereas our study was based on 119 eyes using a different Amaris platform. The differences between studies may result from different patient selection criteria rather than different platforms. When analyzing eyes with mixed astigmatism, 28 % of cases were within ±0.50D of intended refraction in the Allegretto group, in comparison to 42 % of eyes in the Amaris group (*p* = 0.060). Our results to some extent correlate to the study by Alió et al. [13], which reported significant undercorrection of mixed astigmatism using Amaris 500 and Aberration-Free profile. Alió reported 26.9 % of patients being within ±0.50D of the attempted correction, and 65.3 % being within ±1.0D. Stonecipher et al. [5] reported the opposite outcome on Allegretto 400Hz, with 100 % of patients having ≤0.50D of residual astigmatism with *r* ^2^ values >0.98.

### High-order aberrations

There was no significant change in high-order aberrations in eyes with myopic astigmatism. This finding supports the definition underlying the aspheric profiles that were designed to keep high-order aberrations of the eyes unchanged after photoablation. Arbelaez et al. [8] found a statistically significant increase in high-order aberrations after myopic astigmatism treatment on Amaris 500, which was in correlation with the amount of refractive error treated. However, the amount of induced aberrations was lower than that from conventional treatment [15]. Stonecipher et al. [6] did not find a change in high-order aberration in myopic astigmatism up to 3.0D using wavefront optimized profile from Allegretto Eye-Q. Our data confirm the earlier findings using the Allegretto profile, but not the findings using the earlier Amaris profile.

In eyes with mixed astigmatism, changes in amount of spherical aberration have been reported with a tendency towards more negative values; however, the changes were below clinical significance [16–18]. We found similar, but statistically significant, trends towards negative values for both lasers, and these support the findings of Alió et al. [13] using the Amaris 500.

The use of a simple single spherical equivalent as an index does not reflect the efficacy and accuracy of compound astigmatism correction. Therefore, separate analyses of refractive outcomes separating sphere, cylinder, and axis would be more reasonable to evaluate surgery for astigmatism. This could also facilitate any nomogram adjustment, with the aim of further enhancing the accuracy of treatment.

Figures 1, 2, 3, and 4 demonstrate that there is a highly significant association between the attempted astigmatic correction, achieved astigmatic correction, and the difference between the attempted and achieved (ΔC). With closer scrutiny of our data, we noticed that residual cylinder stays in the same direction as the original one, with a ±20° change. From that observation, we noticed that both lasers tend to undercorrect the astigmatism. However, in several cases with cylindrical corrections of ≥6.0D, both lasers overcorrected, since we observed that the postoperative axis shifted by near 90°. To scientifically support this observation, vector analysis would be needed [19].

In summary, both lasers produced acceptable results tending to preserve optical performances of the eye without significant induction of high-order aberrations. There is no difference in effectiveness between lasers for spherical correction. However, Schwind Amaris 750S demonstrated better results and less residual cylinder than Wavelight Allegretto Eye-Q. Nevertheless, the correction of both lasers may yield small residual cylinder. Future studies, with more intensive mathematical analysis of astigmatism itself, are needed to further improve formulas and laser nomograms for cylinder correction.
